# ESTuber db: an online database for *Tuber borchii *EST sequences

**DOI:** 10.1186/1471-2105-8-S1-S13

**Published:** 2007-03-08

**Authors:** Barbara Lazzari, Andrea Caprera, Cristian Cosentino, Alessandra Stella, Luciano Milanesi, Angelo Viotti

**Affiliations:** 1 Istituto di Biologia e Biotecnologia Agraria, via Bassini 15, 20133 Milan, Italy; 2 CISI, Via Fratelli Cervi 93, 20090 Segrate (MI), Italy; 3 Parco Tecnologico Padano, Via Einstein – Località Cascina Codazza, 26900 Lodi, Italy; 4 Istituto Tecnologie Biomediche, Via Fratelli Cervi 93, 20090 Segrate (MI), Italy; 5 Darmstadt University of Technology, Institute of Botany, Schnittspahnstrasse 3-5, 64287 Darmstadt, Germany

## Abstract

**Background:**

The ESTuber database () includes 3,271 *Tuber borchii *expressed sequence tags (EST). The dataset consists of 2,389 sequences from an in-house prepared cDNA library from truffle vegetative hyphae, and 882 sequences downloaded from GenBank and representing four libraries from white truffle mycelia and ascocarps at different developmental stages. An automated pipeline was prepared to process EST sequences using public software integrated by in-house developed Perl scripts. Data were collected in a MySQL database, which can be queried via a php-based web interface.

**Results:**

Sequences included in the ESTuber db were clustered and annotated against three databases: the GenBank nr database, the UniProtKB database and a third in-house prepared database of fungi genomic sequences. An algorithm was implemented to infer statistical classification among Gene Ontology categories from the ontology occurrences deduced from the annotation procedure against the UniProtKB database. Ontologies were also deduced from the annotation of more than 130,000 EST sequences from five filamentous fungi, for intra-species comparison purposes.

Further analyses were performed on the ESTuber db dataset, including tandem repeats search and comparison of the putative protein dataset inferred from the EST sequences to the PROSITE database for protein patterns identification. All the analyses were performed both on the complete sequence dataset and on the contig consensus sequences generated by the EST assembly procedure.

**Conclusion:**

The resulting web site is a resource of data and links related to truffle expressed genes. The Sequence Report and Contig Report pages are the web interface core structures which, together with the Text search utility and the Blast utility, allow easy access to the data stored in the database.

## Background

Despite being dependent on other living organisms for energy supply, fungi have colonized every biotic or abiotic environment. Deciphering their genetic information is important *per se *but also provides integrative knowledge on the genetic interactions among fungi and other organisms. In this work the hypogeous symbiotic white truffle *Tuber borchii (Tb) *was considered. This species has recently attracted attention for its *in vitro *manipulation properties [1], as a possible resource of antiviral agents [2] and for other interesting features [3,4].

A cDNA library was prepared from *in vitro *cultured mycelia and extensive EST sequencing was performed. A total of 2,389 successful sequences was obtained and submitted to the EBI databank for GenBank accession number assignment. Sequences were given the accession numbers from AM165128 to AM167516. Additional 882 sequences from *Tb *vegetative hyphae and ascocarps were downloaded from GenBank and added to the dataset, thus obtaining a collection of 3,271 *Tb *ESTs extracted from five libraries. The aim was to produce an extensive and easily accessible database for truffle, providing sequence analysis and annotation, as well as links to related resources. To date, the ESTuber db [5] is the most complete information repository for truffle EST sequences. Together with the truffle.org project databases [6], ESTuber db can offer information of wide interest to truffle investigators.

## Construction and content

### Library preparation and clone management

*Tb *vegetative hyphae were grown on PDA agar medium for 20 days, collected and lyophylized. Total RNA was extracted by lithium chloride differential precipitation and poly(A)^+ ^RNA was purified on a cellulose oligo-dT column. cDNA was synthesized using the Stratagene's (La Jolla, CA) cDNA synthesis and cloning kit, directionally ligated into the Uni-zap XR (λ zap II) vector (site 1, EcoRI; site 2, XhoI) and packaged using Gigapack Gold packaging extracts. A library (named 'T' in the ESTuber db web interface) was obtained representing more than 7 × 10^7 ^plaque forming units. Plasmids (pBluescript SK) containing cDNA inserts were mass-excised from phage stocks using ExAssit Interference Resistant Helper Phage and propagated in SOLR cells, according to the manufacturer's instructions.

More than 12,000 plasmid clones were selected and replicated twice in 384 well microplates by the colony picking QPix workstation (Genetix). 3,068 clones were subsequently amplified in 96 well microplates by the TempliPhi Amplification kit (Amersham), then lyophylized and submitted to Macrogen Inc. (Seoul, Korea) for direct 3' sequencing. Sequencing was performed with the BigDye Terminator method (Applied Biosystems) on capillary sequencers, using the M13 reverse primer.

### Sequence introduction in the database

From sequencing of the T library, a total of 3,068 sequences was obtained. Electropherograms were read with the Phred program [7] and multifasta sequence and quality files were created and used to feed the program Lucy [8]. Both low quality regions and vector sequences were removed, according to Lucy standard parameters, thus obtaining 2,389 successful sequences longer than 100 bp. As 3' sequencing was performed, vector-free, high quality sequences were reverse-complemented with the program REVSEQ [9].

882 *Tb *sequences from two non-normalised ascocarp libraries (named 'MF' and 'IF' in the web interface, obtained from mature and immature ascocarps, respectively) [10] and from two non-normalised mycelium libraries (named 'SM' and 'LM', obtained from vegetative hyphae grown on solid or liquid medium, respectively) [11], were downloaded from GenBank and added to the dataset. Full details on the five libraries are included in the Library details page of the ESTuber web site. All the sequence files, the corresponding GenBank accession numbers and the available quality files and electropherograms were stored in the MySQL database.

### Sequence processing and annotation pipeline

**Figure 1:**
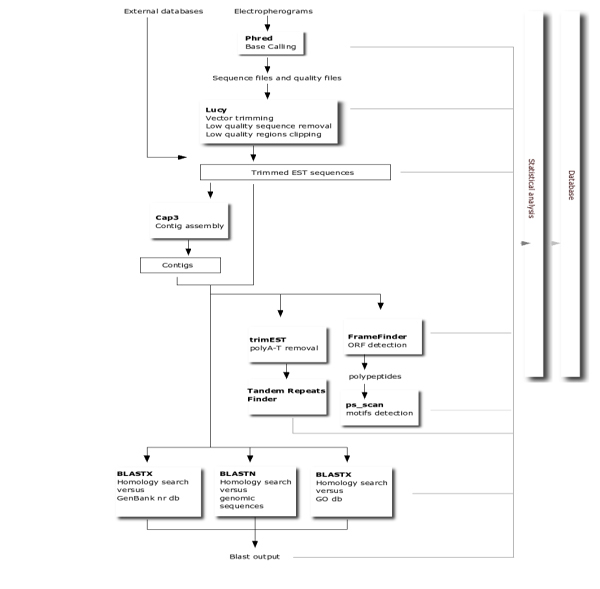
**The ESTuber db pipeline**. Data flow among the main programs included in the ESTuber db pipeline. Accessory programs were excluded from the chart.

An automated pipeline (Fig. 1) was prepared to process EST sequences using public software integrated by a number of in-house developed Perl scripts. The complete dataset in multifasta format was used as input for the CAP3 program [12]: parameters were set to -p 98 -o 100 -f 30, for appropriate EST clustering. The CAP3 output was parsed and relevant data were stored in the database. A unigene dataset was defined, including all the singlets and each contig's longest sequence.

The 3,271 EST sequences and all the contig consensus sequences were annotated locally with a triple procedure.

The main annotation was performed with blastx [13] versus the GenBank nr database (referred to as 'NCBI blast' in the web interface). The GenBank nr database encompasses all non-redundant GenBank CDS translations + RefSeq Proteins + PDB + SwissProt + PIR + PRF for a total of more than 2,450,000 sequences, at present. All possible frames were considered for EST translation. Low complexity filters were disabled, while the remaining blastx parameters were the defaults and no threshold was set. The BLAST output was parsed with an in-house prepared parser. The most important BLAST output values, as well as the complete BLAST output pages, were stored in the database.

The second annotation (referred to as 'genomic blast' in the web interface) was performed against an in-house prepared database encompassing 42,029 fungal genomic sequences from four filamentous fungi (*Aspergillus *ssp, *Fusarium *ssp, *Magnaporthe *ssp, *Neurospora *ssp) and a dimorphic fungus (*Saccharomyces *ssp), downloaded from the GenBank CoreNucleotide db (January 2006). Complete chromosome sequences as well as whole genome shotgun sequences were excluded from the database. The blastn algorithm was used, low complexity sequence filtering was performed and the E-value threshold of 1e^-10 ^was set.

The third annotation was performed with blastx versus the UniProtKB database [14] – encompassing at present more than 2,700,000 entries – that is the reference database for the Gene Ontology (GO) project [15] (referred to as 'GO blast' in the web interface). The E-value threshold of 1e^-10 ^was adopted.

Based on data contained in the available database of associations among proteins and GO elements [16], a Perl script was developed to relate ESTuber GO blast best blast hits to Gene Ontology categories. All the GO elements related to each best blast hit protein were considered, even those having indirect relations. In these cases, the GO 'is_a' relations were taken into account. Matching ontologies were stored into the database, to allow successive dynamic creation of statistics for the ontology occurrences. 130,459 EST sequences from five fungi (the same organisms considered for the preparation of the genomic blast database) were downloaded from GenBank and processed for GO annotation to provide intra-species comparisons of the ontology distribution in GO categories.

The annotation procedures were carried out either on a Linux 9-biprocessor AMDx64 cluster with 2 GB RAM on each node with the mpiBLAST program [17], that allows parallelization of the BLAST procedure, or through the BioinfoGRID [18] computing resource, that uses a series of calculation sites to perform very time-consuming elaborations in a distributed way, parallelizing their executions and exploiting the use of remote resources efficiently.

The entire EST dataset, as well as the contig consensus sequences, were processed for tandem repeat search with the Tandem Repeats Finder program (parameters: 2, 7, 7, 50, 2000) [19]. Prior to repeat search PolyA and PolyT tail containing sequences were trimmed with the TrimEST program (EMBOSS) (parameters: -minlength 4 -mismatches 3). Links to the Tandem Repeats Finder output files were introduced in the web interface, when present.

The ESTate package programs [20] were used to infer putative protein sequences from the whole ESTtuber dataset and from the contig consensus sequences. Fasta2usage (parameters: -w 6 -j 3) and wordprob were used to prepare specific word probability files and FrameFinder was used for sequence translation and selection of the most probable putative protein sequences. During the protein prediction procedure, comparisons were made among protein sets obtained with the same parameters but with different word probability files. As a very limited number of *Tuber *coding sequences was present in public databases, there was no possibility to generate a truffle specific word probability file. Two different in-house generated files were tested: the former obtained from 111,936 fungi mRNA sequences downloaded from the NCBI RefSeq database, and the latter specific for filamentous fungi, obtained from 41,190 mRNA sequences from *Magnaporthe *ssp, *Aspergillus *ssp and *Neurospora *ssp, downloaded from RefSeq. No *Tuber *ssp and *Fusarium *ssp mRNA sequences were present in RefSeq to date. Comparisons on the derivative protein datasets were based on sequence length and on the number of sequences finding their best translation on a lower frame, with respect to the expected composition of forward/reverse oriented sequences of the dataset. When considering these parameters, the best results were obtained with the first of the above-mentioned word probability files. Furthermore, the number of PROSITE matches found for the protein set created according to the fungi specific word probability file was 10% higher than the one retrieved when translations generated according to the filamentous fungi specific file were used. A supplementary control on the automatic protein prediction procedure was carried out by BLAST. Contig consensus sequences were annotated by blastx against the GenBank nr database and six frames translations were considered. Translations of the contig consensus sequences obtained by FrameFinder were also annotated by blastp against the same database and results were compared. Differences were observed for 15 sequences out of 356 (4.2%), that were annotated on a different translation frame with respect to the one selected by FrameFinder. Based on these observations, the FrameFinder algorithm was considered suitable for this work purposes, and protein sequences predicted according to the fungi word probability file were inserted in the database and used as queries against PROSITE [21].

Comparisons to the PROSITE database – version as of 24/01/2006 – were performed locally with the ScanPROSITE program [22] and matching patterns were retrieved and linked to the corresponding sequences in the web interface. Frequently matching patterns and profiles were excluded from the analysis.

Further details on software usage and parameters setting are provided at the Processing, Assembly and Annotation Protocol page of the cited web site.

Statistics on the sequence analysis and on the database current status were inferred from data stored in the database and are reported in Table 1.

### The database

The ESTuber MySQL database is a dynamic structure where new sequences will be added and new features are being implemented, so the database structure is subject to modifications.

**Figure 2:**
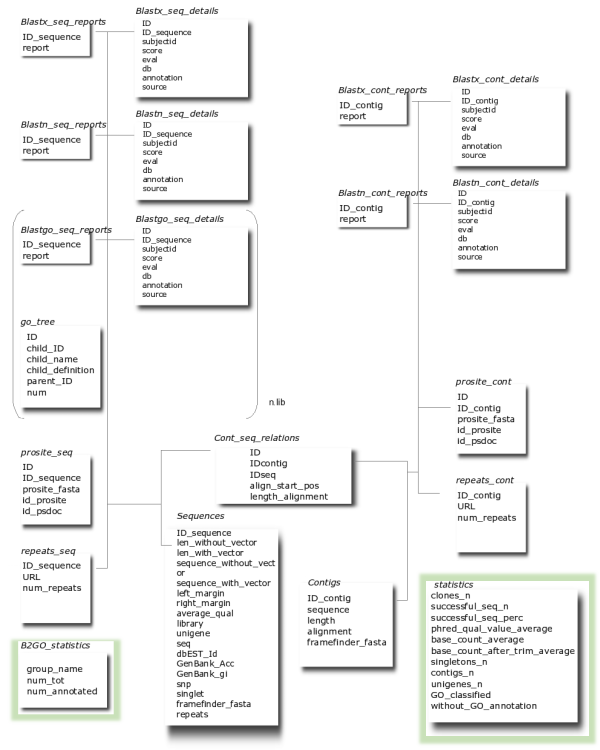
**The ESTuber database**. Main tables of the ESTuber db.

The 'Sequences' table and the 'Contigs' table are the core structures of the database, together with the 'Cont_Seq_relations' table, where relations among sequences and contigs are defined (Fig. 2).

### The web interface

The ESTuber db web interface is based on the php language and manages all the incoming queries as well as all the graphical outputs dynamic creation.

**Figure 3:**
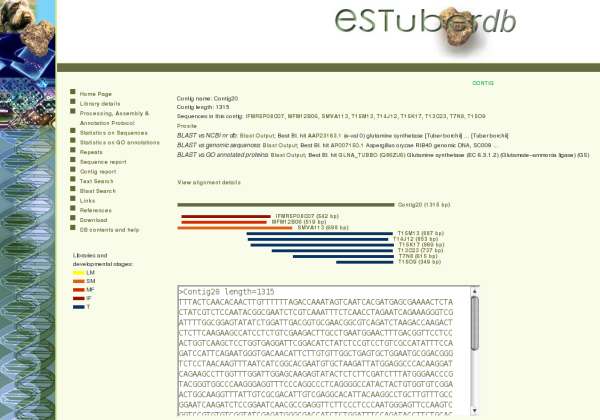
**The ESTuber db contig page**. A detailed page for each contig is included in the database, where a graphical display, the contig consensus sequence and the contig alignment are given, together with any other supplementary information.

Contig graphical display and GO statistics graphical bars are prepared on-the-fly in response to the users' requests and are not stored in any part of the database (Fig. 3). The dynamic management of these data provides up-to-date displays without the need for refreshing corresponding database tables.

## Utility and discussion

### Data flow in the ESTuber db pipeline

Sequence introduction in the ESTuber db was kept apart from the main automated pipeline as chromatograms or sequence quality files were not always available. EST sequences downloaded from public repositories lack all the upstream information and were introduced as such. Nonetheless, when possible, quality data were stored in the database for possible further analysis with programs that require these data as mandatory.

The main pipeline is fed with a single multifasta file containing all the available EST sequences; a number of in-house developed Perl scripts allows automatic data flow among the main programs ensuring full compatibility among all the programs' input/output files. Program outputs are parsed and all relevant data are stored in the database, so that at the end of the pipeline the database tables are completely filled in. The pipeline structure is modular: each main program can be added or removed from the analysis easily and quickly. This allowed the authors to use this same structure for different purposes, with different datasets ([23] and A. Caprera, B. Lazzari, A. Stella, P. Mariani, unpublished results).

### Data availability and retrieval

The ESTuber db was structured to be a repository of data obtained from EST sequence analysis and to provide links to related external resources. Three main interfaces were set up to access the database contents: the Sequence/Contig report pages, the Text search utility and the local Blast utility.

The Sequence report and Contig report pages are the starting points to access sequence and contig related data. Data are presented in summary tables and are linked to internal and external sources. Furthermore, each sequence and each contig were assigned a detailed page where the nucleotide sequence is given. In each contig page, the contig graphical display is provided, as well as the contig alignment. Original blast outputs are retrievable for each sequence and each contig consensus sequence.

The text search utility is structured to allow performing combined keyword searches on all the fields of the Sequence/Contig report tables. As NCBI blast results are presented for each sequence, a user-defined significance threshold based on the E-value can be set in the search to retrieve only significantly annotated sequences. Searches can be restricted to sequence subsets (unigenes/not unigenes, singlets/contig related sequences or repeats containing sequences). Query outputs have the same format of the sequence/contig report tables, presenting only the entries matching the searched query, and can be downloaded both as multifasta sequence files and as text files with tab separated values, containing all the Sequence/Contig report page fields. This format can be easily imported in the most common spreadsheets.

The local blast interface allows users to blast their own sequences against either the nucleic ESTuber db dataset or the derivative putative protein database.

Additional information on the sequence dataset is provided by the two statistics web pages: the first reporting statistics on sequences and on the database status, and the second on the ontologies distribution in the database. GO blast annotations were used to infer the distribution of the annotated sequences among Gene Ontology functional categories, and statistics on the ontology occurrences were created for all the sequences of the dataset, for library-specific subsets and for the mycelium and ascocarps subsets. Additional statistics are provided concerning a dimorphic and four filamentous fungi EST datasets. Ontologies can be hierarchically browsed and are extensively linked to the AmiGO web pages [24].

Download of the ESTuber sequences and of the contig consensus sequences is allowed in multiple formats, and CAP3 alignments and .ace files are also available. Sequence user-defined subsets can be easily downloaded as query outputs.

The web interface includes a detailed help page to assist users in browsing the ESTuber db and in interpreting program outputs.

Sequence annotations are periodically updated and the consistency of external links is constantly verified. We report here on the second version of the ESTuber db: the previous release, including only the T library sequences, is maintained and is accessible via the link given in the ESTuber home page.

### Dataset analysis

The ESTuber db unigene dataset consists of 2,427 sequences. Based on sequence annotation versus the GenBank nr db, 1,400 *Tuber borchii *unigenes were related to known sequences (BLAST E-value = 1e-10). Therefore, the remaining 1,427 unigenes are novel sequences. Among these, the 73.16 % belong to the in-house prepared mycelium cDNA library.

The assembly procedure generated 356 contigs. Analysis of their composition revealed that only 25 contigs contain both ascocarp and mycelium sequences, while the others are tissue-specific, 269 being composed only by mycelium sequences and 62 being composed only by ascocarp sequences. The most abundant transcripts in the database are assembled in four contigs, two composed by ascocarp sequences (Contig 86, 35 sequences, and Contig 41, 26 sequences) and two by mycelium sequences (Contig 55, 28 sequences, and Contig 210, 25 sequences). None of these contigs is significantly annotated, thus confirming the specificity of these sequences for truffle.

### Tandem repeats analysis

Tandem Repeats Finder identified 250 repeats-containing sequences in the complete EST dataset as well as 32 repeats-containing sequences in contig consensus sequences. Due to the fact that a number of EST sequences contained more than one repeat, 331 repeats were actually identified in the complete dataset. The same analysis was performed on a dataset including all the singlet sequences and the contig consensus sequences, and 33 repeats longer than 24 basepairs were identified. Blastn analysis of these repeats against the nr nucleic GenBank database (parameters: -w 7 -e 1000) did not identify any significant homology with regions associated to known features. The repeats consensus sequences were translated with Transeq (EMBOSS) and the outcoming aminoacid sequences were used to scan the PROSITE database. Three known patterns were identified: the ubiquitin domain signature and profile (PROSITE entries PS00299 and PS50053, found in Contig22), the bipartite nuclear targeting sequence (PROSITE entry PS00015, found in sequence T15O19) and the ankyrin repeat region circular profile (PROSITE entry PS50297, found in sequences T12B4 and SMVA56).

The Tandem Repeats Finder algorithm was conceived to identify tandem repeats, but due to the fact that short sequence repetitions are considered as contiguous repeats with coinciding period size and repeat size, the algorithm identifies simple sequence repeats (SSR), too. In the ESTuber db sequence collection, the repeats consensus sequence length varied from 1 basepair (single base stretches), to 230 basepairs (in the polyubiquitin sequence T10J12), and 51 repeats (15.4 % of the total) were SSR, with period sizes shorter than 5 basepairs.

### Protein prediction and protein domains search

When PROSITE was queried with the ESTuber putative protein dataset, 551 positive hits were recovered, while 111 hits were reported when PROSITE was queried with contig consensus sequences translations. All the matching patterns were introduced in the ESTuber db and links to the correspondent PROSITE entries were added.

### Ontologies distribution analysis

Statistics on the *Tuber *ontology occurrences are provided for the whole sequence dataset, for library-specific subsets and for the ascocarps and mycelia subsets. Data from two libraries (T and SM), both obtained from hyphae grown on solid medium, are also merged in a further subset.

**Figure 4:**
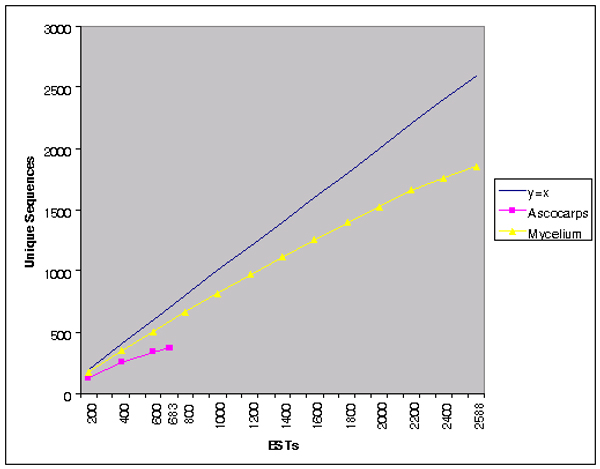
**Discovery rates for the ascocarp and mycelium datasets**. Organ unique sequences plotted against the number of ESTs. The discovery rate is represented by the angular coefficient of the line connecting two consecutive points. The straight line (y=x) corresponds to a discovery rate of 1.

Although single library contribution to the whole dataset is variable, the T library being far more represented than the others, the GO annotation percentage varies from 57.68 in the MF library to 46.84 in the T library. The NCBI annotation results reflect the same trend, the ascocarp libraries being more annotated than the mycelium ones and spanning from 70.33% of annotation in the MF library to 57.47% of annotation in the T library (data available on the ESTuber web site). Furthermore, ascocarp libraries are more redundant than mycelium libraries, as evident from the unigene percentage which is much lower for ascocarp sequences (62,22%) than for mycelium sequences (84,89%). This is confirmed by the analysis of the discovery rates obtained for the pools of ascocarp and mycelium sequences (Fig. 4).

Changes in the distribution of the ontology occurrences between sequences from ascocarps and from cultured hyphae are evident mainly in GO categories related to biological processes (Table 2). These differences are also present in comparisons between sequences from ascocarps at different maturation stages, the 'biological process' GO category being over-represented in mature ascocarps with respect to immature ascocarps (data available in the 'Statistics on GO annotation' page of the database web site).

Statistical analysis of the ontologies distribution is provided also for other five organisms (Tab. 2). Four of these (*Aspergillus*, *Fusarium*, *Magnaporthe *and *Neurospora*) are classified as filamentous fungi, while the fifth, *Saccharomyces*, has a dimorphic behaviour, exhibiting both yeast forms and pseudohyphal phases. Dimorphism in *S. cerevisiae *is associated with the expression of specific proteins [25]. *Saccharomyces *EST sequences were considered in the ontologies analysis, to investigate for a possible differential distribution of ontologies occurrences with respect to the other monomorphic filamentous fungi.

With the aim to investigate the redundancy level of the different EST collections, species-specific assembly procedures were performed with CAP3 and the number of unigenes was determined for each dataset (Tab. 2). CAP3 parameters were the same as in the ESTuber pipeline.

**Figure 5:**
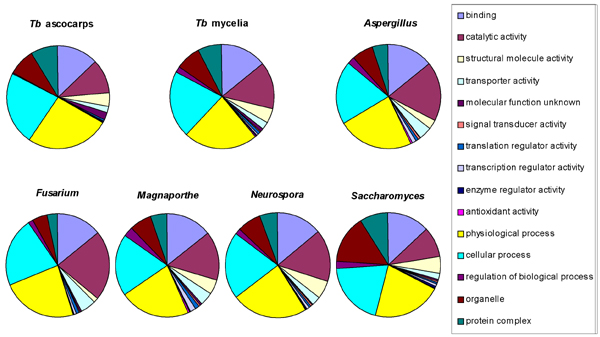
**Pie representation of the ontology occurrences**. Distribution of the matching ontologies in the second-level GO categories for the different considered organisms. Only GO categories where at least one of the organisms had a percentage of matches > 1 were included in pies.

Comparisons among different fungi EST collections reveal a very high GO annotation percentage of *Saccharomyces *sequences. Furthermore, the *Saccharomyces *dataset is mostly composed of unigenes. Actually, *Aspergillus *and *Saccharomyces *are more represented than the other fungi in the UniProtKB database, while *Magnaporthe *is under-represented. As a consequence, the high annotation percentage of *Saccharomyces *sequences could be almost in part attributed to the comparison database composition. Pie graphical representation of the ontology occurrences in the different organisms provides a presentation of the data that is independent from the total annotation percentage (Fig. 5). Accordingly, differences in representation of specific GO categories are evident among the presented organisms.

## Conclusion

The ESTuber db version as of January 2006 encompasses 3,271 EST sequences from ascocarps and from *in-vitro *cultured hyphae. At the moment none of the represented libraries is specifically oriented to the study of the processes that regulate the establishment of symbiosis between the host plant and the fungus. This is actually the most interesting aspect of the truffle project itself. As truffle is a typical niche product the interest in its biology is concentrated in the few producing countries, but a deep insight in the mechanisms involved in the symbiosis establishment could be of wider interest. The efforts of the truffle research community will, hopefully, lead to the production of new sequences and other related data. Even if at the moment the limited number of sequences doesn't allow any other significant analysis, the ESTuber db is structured to be easily integrated with new functionalities. New tools will be added in response to the future needs of the project.

## Availability and requirements

The ESTuber db is available at .

## Authors' contributions

BL prepared the T library, defined the pipeline structure and parameters and drafted the manuscript. AC structured the database and the web interface and wrote all the pipeline accessory programs. CC cared about clone and sequence management. AS participated in the design of the study and critically revised the manuscript. LM coordinated the integration of bioinformatical resources. AV guided and coordinated the execution of the project. All authors read and approved the final manuscript.

## Figures and Tables

**Table 1:** Statistics on the ESTuber db status. Sequences are considered annotated for Best Blast Hit E-values ≤ 1e^-10^.

Total number of sequences	3,271
Average base count	530.43
Number of singletons	2,071
Number of contigs	356
Number of putative unigenes	2,427
Sequences annotated *versus *GenBank nr db (%)	60.19
Sequences annotated *versus *GO db (%)	48.88
Sequences annotated *versus *genomic sequences db (%)	8.6
Repeats-containing sequences (%)	7.64
Sequences containing PROSITE matches (%)	16.84

**Table 2:** Ontologies distribution in GO categories.

	*Tb *ascocarps	*Tb *mycelia	*Aspergillus*	*Fusarium*	*Magnaporthe*	*Neurospora*	*Saccharomyces*
Total number of sequences	683	2,588	35,924	10,302	53,102	28,089	3,041
Unigenes percentage	62.22	84.89	91.6	91.67	94.78	56.28	99.44
GO annotation percentage	55.49	46.14	61.09	46.89	44.54	44.15	78.72
							
**MOLECULAR FUNCTION**	39.97	42.19	54.2	43.74	40.77	40.14	67.91
binding	20.94	21.14	25.41	19.78	18.84	19.5	43.47
catalytic activity	17.72	20.83	32.1	30.35	20.39	21.98	30.71
structural molecule activity	6.59	6.45	5.11	2.3	5.57	7.77	16.38
transporter activity	3.51	3.71	6.43	6.57	5.76	3.9	6.91
molecular function unknown	3.37	0.97	0.88	0.62	0.72	0.54	1.84
signal transducer activity	0.15	0.39	1.05	0.49	0.6	0.61	0.72
translation regulator activity	1.02	1.43	1.2	1.48	1.82	0.95	1.51
transcription regulator activity	0	0.46	1.79	0.79	2.39	1.08	2.7
enzyme regulator activity	0.15	0.7	0.39	0.19	0.34	0.2	1.71
antioxidant activity	0.73	0.35	0.62	0.14	0.55	0.48	1.25
							
**BIOLOGICAL PROCESS**	42.02	35.55	43.59	34.49	32.45	34.18	68.33
physiological process	41.43	32.92	40.93	32.93	28.91	32.4	67.41
cellular process	37.92	30.87	35.44	30.36	25.61	28.93	66
regulation of biological process	0.73	2.09	3.7	1.96	4.04	2.21	6.68
							
**CELLULAR COMPONENT**	15.96	15.92	15.56	8.83	11.89	12.84	55.87
organelle	13.76	11.67	11.81	6.88	8.97	10.5	50.51
protein complex	13.91	11.01	8.79	4.46	7.11	7.73	28.74

Ontologies distribution in the three main GO categories and in their subclasses of the *Tb *ascocarps and mycelia subsets and of the five fungi EST datasets considered for comparison purposes. Data on EST collection redundancies and on the GO annotation percentages are given at the top of the table. Matches with GO entries are expressed as percentage of matching sequences with respect to the total number of sequences of the considered dataset. Only GO categories where at least one of the considered organisms had a percentage of matches > 1 were included in the table.
